# Supplementary material for: SynergyFinder Plus: Toward Better Interpretation and Annotation of Drug Combination Screening Datasets
Source: Genomics Proteomics Bioinformatics. 2022 Jan 25;20(3):587–96. doi: 10.1016/j.gpb.2022.01.004 (PMC9801064; doi:10.1016/j.gpb.2022.01.004)
Supplement: Supplementary Table S1 [file mmc1.docx]

**Table S1 The key differences in the mathematical modeling between SynergyFinderPlus and SynergyFinder2 for three-drug combinations**

|  | SynergyFinderPlus | SynergyFinder2 |
| --- | --- | --- |
| BLISS | $S_{BLISS}=E_{A,B,C}-{(E}_{A}+E_{B}+E_{C}-E_{A}E_{B}-E_{A}E_{C}-E_{B}E_{C}+E_{A}E_{B}E_{C}$) | $S_{BLISS}=E_{A,B,C}-{(E}_{A}+E_{B}+E_{C}-E_{A}E_{B}-E_{A}E_{C}-E_{B}E_{C}-E_{A}E_{B}E_{C}$) |
| LOEWE | $S_{LOEWE}=E_{A,B,C}-E_{LOEWE},$  $s.t.\sum_{i\in\{A,B,C\}} (\frac{x_{i}}{f_{i}^{-1}(E_{LOEWE})})=1$ | $S_{LOEWE}=\frac{a}{A}+\frac{b}{B}+\frac{c}{C}$ |
| ZIP | $S_{ZIP}=\hat{E}_{A,B,C}-\left( \hat{E}_{A}+\hat{E}_{B}+\hat{E}_{C}-\hat{E}_{A}\hat{E}_{B}-\hat{E}_{A}\hat{E}_{C}-\hat{E}_{B}\hat{E}_{C}+\hat{E}_{A}\hat{E}_{B}\hat{E}_{C} \right)$  $\hat{E}_{A,B,C}=\frac{1}{3}(\frac{\hat{E}_{-A}+\left( \frac{x_{A}}{\hat{m}_{A}} \right)^{\hat{\lambda}_{A}}}{1+\left( \frac{x_{A}}{\hat{m}_{A}} \right)^{\hat{\lambda}_{A}}}+\frac{\hat{E}_{-B}+\left( \frac{x_{B}}{\hat{m}_{B}} \right)^{\hat{\lambda}_{B}}}{1+\left( \frac{x_{B}}{\hat{m}_{B}} \right)^{\hat{\lambda}_{B}}}+\frac{\hat{E}_{-C}+\left( \frac{x_{C}}{\hat{m}_{C}} \right)^{\hat{\lambda}_{C}}}{1+\left( \frac{x_{C}}{\hat{m}_{C}} \right)^{\hat{\lambda}_{C}}}$) | $S_{ZIP}=E_{A,B,C}-\left( \hat{E}_{A}+\hat{E}_{B}+\hat{E}_{C}-\hat{E}_{A}\hat{E}_{B}-\hat{E}_{A}\hat{E}_{C}-\hat{E}_{B}\hat{E}_{C}-\hat{E}_{A}\hat{E}_{B}\hat{E}_{C} \right)$ |

*Note*: BLISS, Bliss independence; LOEWE, Loewe additivity; ZIP, zero interaction potency.
